# Ultra Low-Conductivity Water by Electrophoretic Ion Exclusion

**DOI:** 10.6028/jres.064A.053

**Published:** 1960-12-01

**Authors:** Wolfgang Haller, H. C. Duecker

## Abstract

Ultra low-conductivity water has been prepared by recirculating it through an electric field of 1,000 volts/centimeter maintained between two ion selective membranes. It was possible to obtain consistently water with an electrical conductivity below the lowest values reported in the literature. The lowest conductivity value which could be reached was 0.039 × 10^−6^ ohm^−1^ cm^−1^ at 18 °C, indicating an ionic impurity content of only one third of the minimum previously reported. This corresponds to the conductivity of a sodium chloride solution at a concentration of 0.0010 parts per million.

## 1. Introduction

In the course of a kinetic diffusion study the authors were faced with the problem of preparing and maintaining ultra low-conductivity water which was to be circulated across the surface of a solid from which very small amounts of an ionic substance were released. It was considered important to remove continuously the incoming ions and to record simultaneously their flux. The method developed for this purpose was found to maintain the water at an extremely low conductivity level. In view of the fundamental importance of the properties of purest water to the physical sciences and because the method exceeds certain previous practical limits in the purification of water, this account of the method applied and the results obtained is given.

Ultra low-conductivity water has been prepared by distillation, ion exchange, and electrodialysis. The lowest conductivity water reported in the literature was obtained by Kohlrausch and Heydweiller [1]^1^ after 36 consecutive vacuum distillations. The present authors chose a modified electrophoretic procedure as such a method served best the dual functions of ion removal and ion detection as required for the diffusion study. This paper, however, deals strictly with the purification function so that the detection function enters the discussion only to the extent that it appears necessary in describing the performance of the apparatus.

Electrophoretic water purification methods have in the past been used largely for processing of brine to obtain water of potable quality [2 through 6], an application where its use is rather a matter of cost than of ultimate purity. The general method of electrophoretically purifying an ion-containing liquid consists of subjecting it to an electrical field in which the cations and anions move toward their respective electrodes and recovering a portion of the liquid which has been depleted of ionic impurities. The isolation of the ion-depleted liquid is very difficult so that it has become the practice to introduce flow-channeling diaphragms. Such diaphragms can be simple porous separators [7], natural organic membranes [8], or synthetic membranes of preferred anionic or cationic permeability [9, 10, 11]. Because of the use of such membranes, electrophoretic purification procedures have been generally called electrodialysis, even in cases where the ion discriminating function of the membranes is not necessarily a fundamental feature of the purification mechanism.

## 2. Method and Materials

**Figure 1:**
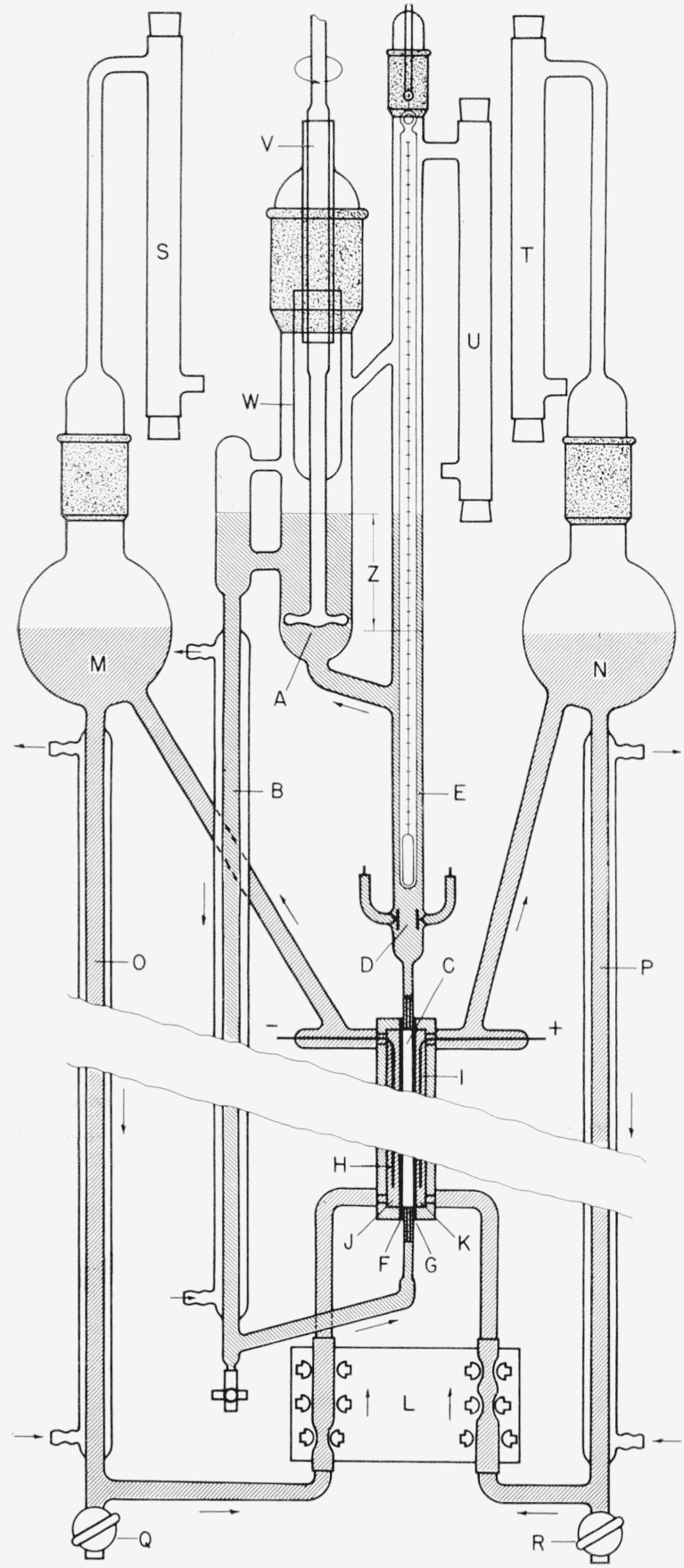
Schematic of water purification apparatus A, Impeller pump; B, heat exchanger; C, center compartment; D, conductivity cell; E, thermometer well; F, cation permeable membrane; G, anion permeable membrane; H, cathode; I, anode; J, cathode compartment; K, anode compartment; L, peristaltic pump; M, cathodal reservoir; N, anodal reservoir; O, heat exchanger; P, heat exchanger; Q,R, drainage stopcocks; S,T,U, breathing tubes; V, glass bearing; W, cup; Z, head produced by pump.

The basic features of the purification apparatus used in this study are illustrated in figure 1. A platinum impeller pump (A) circulates water through a closed loop consisting of a heat exchanger (B), the center compartment of the purification cell (C), the conductivity cell (D), and thermometer well (E). The impeller pump produces a head, or level difference, in the loop which is indicated by (Z) in the drawing. The center compartment of the purification cell is separated from the side cells by a cation permeable membrane (F) and an anion-permeable membrane (G). Electrodes (H, I) supply a d-c voltage to the electrolyte solutions contained in the side compartment of the purification cell. The cathode compartment (J) is filled with 0.05 *N* ammonium hydroxide and the anode compartment (K) is filled with 0.02 *N* sulfuric acid. The anodal and cathodal electrolytes are circulated through separate closed loops by means of a peristaltic action pump (L). The most important purpose of circulating the electrodal liquids is homogenization of the electrolyte concentration in the side compartments of the purification cell. Under the influence of the electric field anionic and cationic impurities migrate from the water in the center cell through the respective membranes, thus depleting the water in the center cell of ionic impurities. The regions of the electrolyte solutions next to the membranes tend to become reduced in their concentration of sulfate and ammonium ions. This tendency, if not counteracted by rapid circulation, would lead to regional increases in the electrical resistance within the side cells, reducing the effective voltage gradient across the center cell. The rapid flow of the electrolyte past the platinum electrodes reduces gas polarization of the electrodes by carrying the gas bubbles into the reservoirs (M, N) where they are allowed to escape. The temperature of the electrodal liquids can be controlled by means of the heat exchangers (O, P).

The main body of the apparatus in contact with the liquids is constructed of borosilicate glass (Pyrex 7740) except the Teflon stopcocks of the drainage valves (Q, R). Tubing connections in the electrodal circuits and the flexible tubing sections through the peristaltic pump are made from plasticized polyvinyl resin (Tygon R-3603). Breathing tubes filled with silica gel and Ascarite (S, T, U) protect the system from the carbon dioxide of the atmosphere.

The water is circulated through the purification cell by means of a motor driven platinum impeller pump. The shaft of the impeller pump enters the system through a precision-bore ground glass bearing (V) which is lubricated with a medium-viscosity petroleum grease. A glass cup (W) sealed to the pump shaft prevents excess lubricant from being splattered into the circulating water.

**Figure 2:**
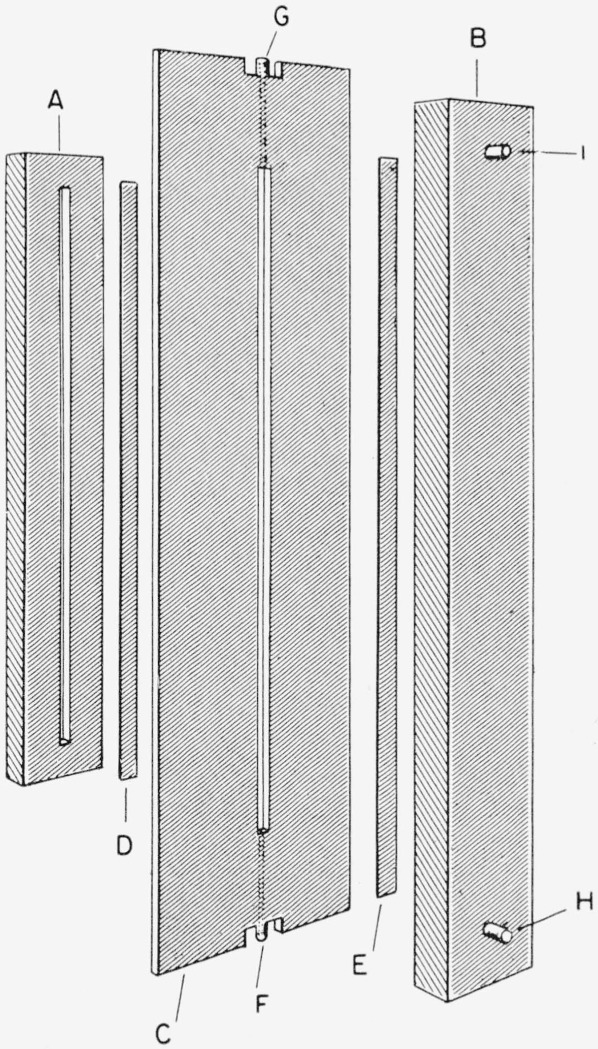
Exploded view of electrophoretic cell A, Cathodal side cell; B, anodal side cell; C, center cell; D, cation permeable membrane; E, anion permeable membrane; F,G, connections to center cell; H,I, connections to side cell.

An exploded view of the electrophoretic cell is given in figure 2. In operation, the two side cells (A, B) are pressed against the center cell (C), thus holding the two strips of ion selective membrane (D, E) in place. The side cells and the center cell are machined from high-density polyethylene sheet. The ion selective membranes are Nalfilm-1 and Nalfilm-2 (manufactured by National Aluminate Corp.). By application of sufficient pressure by means of a clamping device (not shown in the figure), the system is maintained leak-free without gaskets or sealant. The water enters and leaves the center cell through the nipples F and G respectively. The anodal liquid enters the side cell B through nipple H and leaves through I. Similarly the cathodal liquid enters and leaves the side cell A through nipples not visible in the drawing. The center compartment of the electrophoretic cell measures 0.3 cm from membrane to membrane, has a width of 0.6 cm and a length of 30.0 cm.

The electrophoretic cell is operated from a voltage stabilized d-c power supply capable of delivering up to 200 ma between 0 and 1500 v. The cell current and voltage are measured with conventional instruments. Inasmuch as the resistance through the side cells is very much lower than that through the water in the center cell, the voltage drop across the center cell is almost identical to that across the electrodes. With the cell geometry and power supply described above, a voltage gradient of up to 5,000 v/cm can be produced.

## 3. Procedure and Performance

The cell constant of the conductivity cell was determined with 0.01 N potassium chloride solution at 18 and 25 °C using the method described by Daniels [12] and the conductivity values of Jones and Bradshaw [13]. The resistance measurements for the cell calibration were made with a calibrated a-c resistance bridge (General Radio type 650–A) capable of being read to within 0.3 percent. The cell constant was found to be 0.368 cm^−1^ with an error of ±0.5 percent.

The resistance of the water in the conductivity cell was measured with a calibrated d-c megohm resistance bridge (General Radio type 544-BS8) since the polarization effects were found to be negligible. The thermometer above the conductivity cell was calibrated by comparison with a certified standard thermometer. The deviation of the temperature of the water between the electrodes of the conductivity cell and the temperature indicated on the thermometer was investigated by circulating water of constant and high purity through the system while lowering and raising the temperature of the water. Temperature readings were taken at the moment of bridge balance at unaltered bridge settings by approaching the resistance from both above and below. The maximum temperature deviation observed for identical conductivities at the rates of temperature change employed in the experiments was always smaller than 0.15 °C, and at room temperature was only 0.05 °C. The maximum conductivity error introduced by the temperature measurement was calculated to be ±0.5 percent.

A number of precautions were taken to prevent the contamination of the water with organic substances and to reduce the influx of ions from the surface of the glass. Among these precautions were: (1) Before operating the purification apparatus, the glass parts were steamed for over 200 hr in such a way that the steam condensed on the inner surface of the equipment and washed away ions extracted from the glass; (2) the ion-permeable membranes were converted into the acid and hydroxyl forms, respectively, and soaked in successive portions of distilled water over a period of days to remove water-soluble residues; (3) the apparatus was operated several days in advance of an experiment with successive portions of organic-free distilled water to remove further traces of impurities in the apparatus.

**Figure 3:**
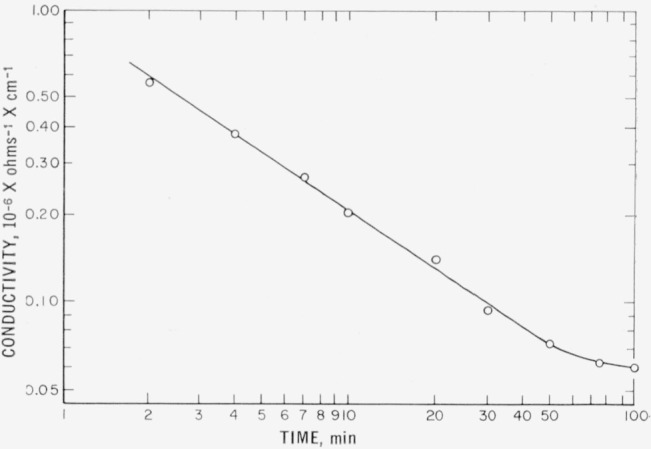
Progress of water purification.

During the purification of distilled water, the electrical conductivity, *k*, of the water in the conductivity cell was found to decrease with time, *t*, according to a power function, *k=at^b^+c*, in which the value of the constant *b* depends on the operational parameters, such as the temperature, the type of contamination in the water, the voltage drop across the membranes, and the flow rates of the water and of the electrolyte. A plot of conductivity versus time in a typical experiment is shown in figure 3, the value of *b* in this case being −0.75. The values of *b* in other experiments were between −0.5 and −0.8. Upon approaching the terminal conductivity, the curve deviates from the power function and approaches a limiting, finite value.

The current consumption of the purification cell depends on the conductivity of the circulated water. Upon introduction of water with a conductivity of about 6×10^−6^ ohm^−1^ cm^−1^, the current through the purification cell assumes within a few seconds a value of about 20 ma at 1,000 v. When the terminal conductivity is reached, the current consumption at 18° is approximately 2.2 ma at 1,000 v and obeys Ohm’s law within 1 percent over the voltage range available. From the dimensions of the purification cell, and the electrical resistance of the cell at the terminal conductivity, a specific conductance of the water in the purification cell can be calculated. The average of the conductivities calculated in this manner from 20 experiments is tabulated in column (a) of table 1. Although the cell constant of the purification cell cannot be very accurately determined, it appears that the water leaving the purification cell is purer than in the conductivity cell.

## 4. Results and Discussion

Using the precautions outlined earlier it takes approximately 1 hr to approach a terminal conductivity after filling the apparatus with a good grade of distilled water. The terminal conductivity of the most successful of 20 experiments is tabulated in column (b) of table 1. Each value is subject to an estimated error of ± 1 percent. Prolonged operation of the apparatus over extended periods will not yield a water of higher purity than obtained after about 2 hrs.

The conductivities of ultra low-conductivity water reported by various researchers are given in table 2. For a better comparison the concentration of a solution of sodium chloride is calculated which would possess a specific conductivity corresponding to the reported values. These equivalent concentrations are given in table 2. The values are calculated using a theoretical conductivity of pure water equal to 0.0371 × 10^−6^ ohm^−1^ cm^−1^ at 18° and 0.0549×10^−6^ ohm^−1^ cm^−1^ at 25°. These values are calculated using the recent tabulations of the ionization constant and equivalent conductivities of the H^+^ and OH^−^ ions given by Robinson and Stokes [23].

Kohlrausch and Heydweiller [1] obtained their water after 36 consecutive distillations in a vacuum and measured its conductivity in a fused silica cell which had been exposed to water for 10 yr. (Attempts by Nernst to reproduce this value were unsuccessful [24]). Kunin and McGarvey [20] report on water from a monobed ion exchange column although the temperature is not given. Those workers who used distillation methods incorporated one or more of the following modifications: (1) Distillation from an alkaline permanganate solution, (2) purging the solution and/or the distillate in the column with an inert gas to remove carbon dioxide, (3) use of materials of construction such as tin or organic substances to reduce mineral content.

The apparatus described by the present authors affords a method to produce water of lower ionic content than any previously reported in the literature. It is expected to be particularly applicable where it is desired to conduct experiments with small quantities (10 to 50 ml) of extremely ion-free water.

## Figures and Tables

**Table 1:** Terminal experimental conductivities

Temperatures	Specific conductivity in 10^−6^ ohm^−1^ cm^−1^		
	(a) Purification cell		(b) Conductivity cell

*°C*	*avg*	*std dev*	*min values observed*
15.0±0.1	……….	……….	0.0330
18.0± .1	0. 038	0.0006	.0390
20.0± .1	……….	……….	.0440
25.0± .05	.057	.0010	.0589
30.0± .1	……….	……….	.0775

**Table 2:** Data on ultra low-conductivity water, obtained by various workers

Worker	Conductivity in 10^−6^ ohm^−1^ cm^−1^	Temperature	Concentr. of a sodium chloride solution in ppm with same conductivity	Method of purification

		°*C*		
This work	$\{\begin{array}{c} 0.0390\\ .0589 \end{array}$	18	0.0010	}Electrophoresis.
		25	.0018	
Kohlrausch and Heydweiller [1]	$\{\begin{array}{c} .0429\\ .062 \end{array}$	18	.0031	}Distillation.
		25	.0032	
Bengough, Stuart, and Lee [14]	.045	18	.0043	Do.
Kraus and Dexter [15]	.048	18	.0059	Do.
Weiland [16]	0.053 to 0.066	18	0.0086 to 0.0156	Do.
Bencowitz and Hotchkiss [17]	.06 to .07	Not given	…………………..	Do.
Thiessen and Herrmann [18]	.065 to .080	25	.0046 to .0118	Do.
Kunin and McGarvey [19]	0.07	Not given	……………………	Ion exchange.
Gostkowski [20]	.07	0	0.0280	Distillation.
Hulett [21]	.081	Not given	……………………	Do.
Bourdillon [22]	.086	18	.0260	Do.
Pure water (theoretical).	$\{\begin{array}{c} .0371\\ .0549\\ .0111 \end{array}$	18	.0000	
		25	.0000	
		0	.0000	

## Footnotes

1 Figures in brackets indicate the literature references at the end of this paper.
